# Thymol and Carvacrol: Molecular Mechanisms, Therapeutic Potential, and Synergy With Conventional Therapies in Cancer Management

**DOI:** 10.1002/fsn3.70936

**Published:** 2025-09-15

**Authors:** Ahmad Mujtaba Noman, Muhammad Tauseef Sultan, Shehnshah Zafar, Muhammad Maaz, Aimen Mazhar, Muzzamal Hussain, Muhammad Imran, Ahmed Mujtaba, Muhammad Tajammal Hussain, Suliman A. Alsagaby, Waleed Al Abdulmonem, Muhammad Asif Khan, Entessar Al Jbawi

**Affiliations:** ^1^ Department of Human Nutrition, Faculty of Food Science and Nutrition Bahauddin Zakariya University Multan Pakistan; ^2^ Department of Food Science Government College University Faisalabad Faisalabad Pakistan; ^3^ Department of Food Science and Technology University of Narowal Narowal Pakistan; ^4^ Department of Food Sciences and Technology, Faculty of Biomedical & Life Sciences Kohsar University Murree Murree Pakistan; ^5^ National Institute of Food Science & Technology (NIFSAT) University of Agriculture Faisalabad Faisalabad Pakistan; ^6^ Department of Medical Laboratory Sciences, College of Applied Medical Sciences Majmaah University AL‐Majmaah Kingdom of Saudi Arabia; ^7^ Department of Pathology, College of Medicine Qassim University Buraidah Kingdom of Saudi Arabia; ^8^ Department of Human Nutrition and Dietetics The University of Lahore Lahore Pakistan; ^9^ Sugar Beet Research Department Crop Research Administration, General Commission for Scientific Agricultural Research (GCSAR) Damascus Syria

**Keywords:** anticancer, apoptosis, carvacrol, cell cycle arrest, PI3K/AKT, thymol, Wnt/ß‐catenin

## Abstract

Monoterpenes like thymol and carvacrol are recognized for their anti‐inflammatory and anticancer properties, predominantly found in the *Lamiaceae* family, particularly in *Thymus* species, but also present in *Verbenaceae, Scrophulariaceae, Ranunculaceae,* and *Apiaceae* families. This review explores their anticancer potential, molecular mechanisms, and synergism with other cancer therapies. The data was collected by using several keywords and MeSH terms, and data from Google Scholar, PubMed, Scopus, and Web of Science indicate that thymol and carvacrol modulate key signaling pathways, including MAPK/ERK, PI3K/AKT, Wnt/β‐catenin, JAK/STAT, HH/GLI, and NF‐κB. They upregulate pro‐apoptotic genes (*Bax*, *Bak*, *Bid*, *p53*, and *SIVA*) while downregulating anti‐apoptotic genes (*Bcl‐2*, *Bcl‐xL*, *XIAP*, and *cIAP1*), leading to apoptosis and cell cycle arrest at G0/G1 and G2/M phases. Thymol derivatives, such as 1,2,3‐triazoles and carvacrol, effectively target breast cancer (BC) through PI3K/AKT/mTOR and NOTCH pathways and inhibit *PIK3CA* expression. In lung cancer (LC), they act as SphK1 inhibitors in NSCLC H1299 and A549 cell lines. Additionally, thymol exhibits anti‐EGFR activity, while carvacrol modulates the HIF‐1α/VEGF pathway, making them potential candidates for colorectal cancer (CRC) management. In vitro studies confirm their efficacy against multiple cancer cell lines (MCF‐7, HT‐29, HeLa, PC‐3, HepG2, HL‐60), while in vivo animal models highlight their antiproliferative and antitumor effects. Their synergistic potential with chemotherapy, radiotherapy, and other bioactive compounds strengthens their therapeutic promise. However, challenges such as stability, bioavailability, and the need for clinical trials hinder their clinical application. This review is the first to comprehensively report thymol and carvacrol in a single study, offering new insights into their anticancer potential.

## Graphical abstract

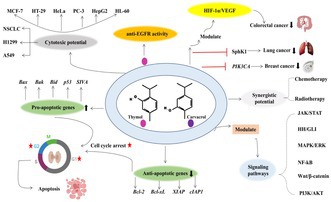

**Abbreviations**
↑: upward arrow
↓: downward arrow
ADHD: attention deficit hyperactivity disorder
ATG5: autophagy‐related protein 5
CDK4: cyclin‐dependent kinase 4
CDK6: cyclin‐dependent kinase 6
CDKs: cyclin‐dependent kinases
CIC: circulating immune complex
CIMP: CpG island methylator phenotype
CIN: chromosomal instability
COX‐2: cyclooxygenase‐2
CPK: creatine phosphokinase
EMT: epithelial‐mesenchymal transition
ESR: erythrocyte sedimentation rate
HH/GLI: hedgehog/GLI
HIF‐1α/VEGF: hypoxia‐inducible factor 1, alpha/vascular endothelial growth factor
IGF‐1: insulin‐like growth factor 1
JAK/STAT: janus kinase‐signal transducer/activator of transcription
JNK: c‐Jun N‐terminal kinase
LDH: lactate dehydrogenase
MAPK/ERK: mitogen‐activated protein kinase/extracellular signal‐regulated kinase
MDA: malondialdehyde
MeSH: medical subject headings
MSI: microsatellite instability
mTOR: mechanistic target of rapamycin
NF‐κB: nuclear factor kappa B
NOTCH: neurogenic locus notch homolog protein
Nrf2/HO‐1: nuclear factor erythroid 2‐related factor 2/transcription factor/heme oxygenase‐1
PI3K/AKT: phosphatidylinositol 3‐kinase/Protein kinase B
STAT3: signal transducer and activator of transcription 3
TGF‐β: transforming growth factor‐beta
TRPM7: transient receptor potential cation channel subfamily M member 7
Wnt/β‐catenin: wingless‐related integration site/catenin beta‐1

## 1 Introduction

Cancer is the leading cause of morbidities and fatalities, affecting ~20 million people in 2022. The overproduction or imbalance of reactive oxygen species (ROS), which are highly unstable and lead to oxidative stress (OS), inflammation, DNA damage, and genetic and epigenetic alterations, which are major risk factors for cancer development. The activation of pro‐inflammatory markers (IL‐6, IL‐1β, TNF‐α, MDA), suppression of tumor suppressor genes (TSGs) like *p53*, *APC*, *CDKN2A*, *BRCA1*, and *BRCA2*, and activation of oncogenes (*ERBB2*, *BCR/ABL1*, *K‐Ras*, *PIK3CA*, *NMYC*) are significant events in oncogenesis (Greten and Grivennikov 2019). Medicinal herbs and bioactive compounds are highly admired worldwide due to their pharmacological properties, such as anti‐inflammation and anticancer, and in the last few decades, their demand has increased (Chopra et al. 2022). The anticancer effects of these herbs are due to versatile bioactive compounds, that is, terpenes, anthocyanins, carotenoids, saponins, and alkaloids (Dubey et al. 2022).

Thymol, also known as 2‐isopropyl‐5‐methylphenol or 5‐methyl‐2‐isopropylphenol (C10H14O), is a monoterpene, colorless, crystalline, with a distinctive odor, and the active ingredient of *T. serpyllum* oil. Besides *T. serpyllum* , it is also present in other varieties of the genus thyme, like *T. vulgaris* , *T. zygis*, *T. praecox* , and oregano of the genus *Origanum*. Thymol is highly soluble in organic solvents but slightly soluble in water at neutral pH (Escobar et al. 2020). Medicinal properties of thymol, such as antioxidant (Yildiz et al. 2021), anti‐inflammatory (Islam et al. 2024), anticancer (Taibi et al. 2024), antidiabetic (Sachan et al. 2022), and hepato‐renal protection (Jamshidi and Taheri 2021; Özmen et al. 2021), have been reported.

**FIGURE 1:**
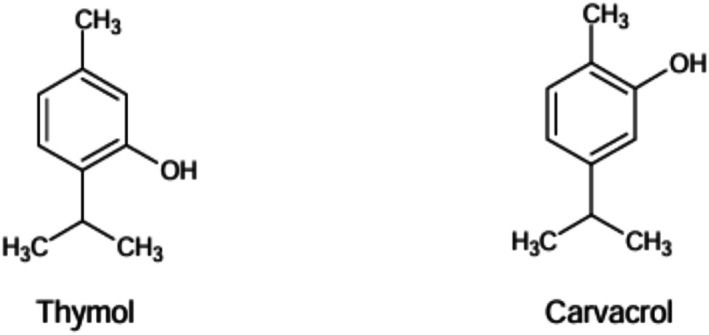
Chemical structures of Thymol (a) and carvacrol (b).

Carvacrol, 2‐Methyl‐5‐(propane‐2‐yl) phenol, an isomer of thymol, a monoterpene also referred to as cymophenol with the chemical formula (C_6_H_3_(CH_3_) (OH)C_3_H_7_). It is insoluble in water while highly soluble in ethanol, CCl4, and diethyl ether. Major sources of carvacrol are *Lavandula multifida*, *Nigella sativa*, *Origanum vulgare*, *Lippia graveolens*, and *Thymus glandulosus* (Imran et al. 2022). The biological properties of carvacrol include antioxidant (Ridaoui et al. 2024), anti‐inflammatory (De Souza et al. 2023), anticancer (Abed et al. 2024), antidiabetes (Hoca et al. 2023), antihypertension (Khazdair et al. 2024), cardioprotective (Joshi et al. 2023), neuroprotective (Forqani et al. 2023) and hepato‐nephroprotective (Cerrah et al. 2023). The chemical structures of both compounds, thymol (a) and carvacrol (b) are illustrated in Figure 1.

The current review covers several aspects of thymol, carvacrol, and cancer management. The review focuses on the anticancer potential of thymol and carvacrol and their possible mechanisms. This review also illuminates mechanisms of anticancer activity like apoptosis induction, cell cycle arrest, and molecular pathways. Moreover, the detailed in vitro and in vivo studies and the synergetic role of thymol and carvacrol with other bioactive compounds, chemotherapeutic drugs, and radiotherapy are the limelight of this review. Lastly, the limitations and challenges, such as the bioavailability of both compounds and strategies to enhance therapeutic activities and clinical trials, are also significant features of this review.

## 2 Search Strategy

**FIGURE 2:**
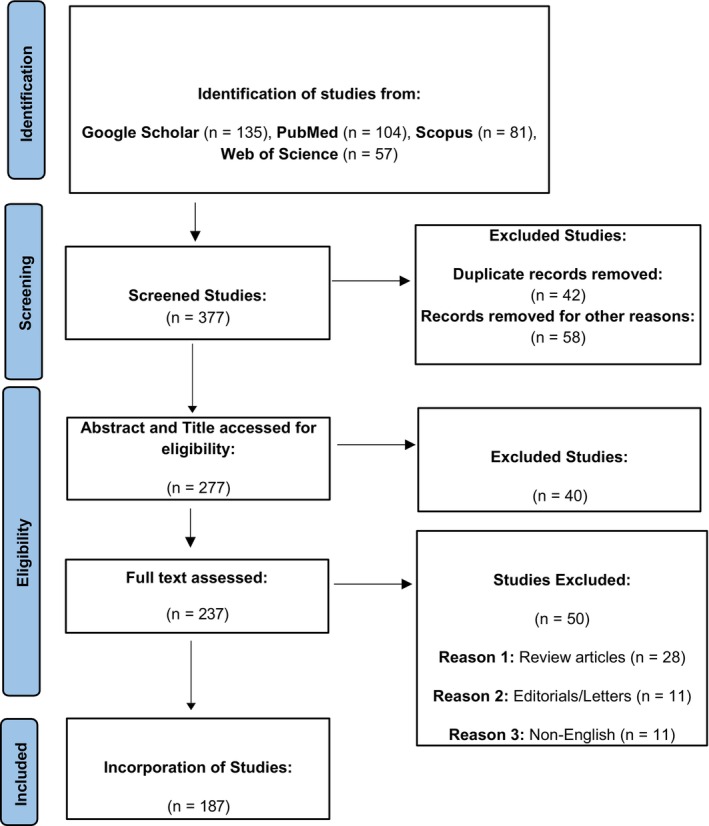
PRISMA flow diagram of the literature search process.

The methodology segment was designed to review the updated anticancer properties of carvacrol and thymol (Lo 2022). Relevant peer‐reviewed articles were retrieved from databases and search engines such as Google Scholar, PubMed, Scopus, and Web of Science, along with keywords and MeSH terms like “Thymol” and “Carvacrol” (e.g., “Thymol and Carvacrol,” “Monoterpenes”), “Anticancer Effects” (e.g., “Antineoplastic Molecules and Agents,” “Cancer Therapy”) and definite cancer types modalities. Moreover, Boolean operators such as AND and OR were used to enhance the search. Inclusion criteria: original research articles inspecting carvacrol and thymol's effects on cancer cells or murine cancer models, studies that explicated molecular mechanisms of carvacrol and thymol's action, and clinical trials evaluating carvacrol and thymol's efficiency. Exclusion criteria: non‐English articles, studies using homeopathic substances, and research including other compounds besides thymol and carvacrol. Data mining focused on the study model, type of cancer, thymol and carvacrol dosage and route of administration, results on cancer cell propagation, apoptosis, cell cycle arrest, and effects on main signaling pathways. The findings were systematically analyzed to evaluate the therapeutic potential of carvacrol and thymol in cancer management. The PRISMA flow diagram of the literature search process is shown in Figure 2.

As shown in the PRISMA flow diagram (Figure 2), 377 studies were found. After removing duplicates and excluding irrelevant papers based on titles and abstracts, 277 articles were retained for full‐text review. Forty articles were excluded after title and abstract assessment, and 50 were excluded after full‐text review, with the reasons for their removal detailed in the PRISMA flow diagram. Finally, 187 studies were included in the review.

## 3 Mechanisms of Anticancer Activity

Studies have demonstrated that anticancer agents act on multiple targets, and their anticancer activity includes several mechanisms of action, such as apoptosis induction, cell cycle arrest, modulation of OS, anti‐metastasis, inhibition of angiogenesis, immune‐modulatory impact, modulation of different pathways, and enzymatic activity (Tang et al. 2022).

### 3.1 Induction of Apoptosis

Apoptosis is a critical factor in programmed cell death, vital for the developmental process and maintaining cellular homeostasis. It is a Greek word that refers to the fall of the leaves during autumn, achieved through external and internal factors (Singh et al. 2022). Studies on thymol have demonstrated its potential role in apoptosis through 2D and 3D techniques. Concerning this, a 2D and 3D (spheroids) murine mammary tumor cancer model, including monolayer 4 T1 cells and L929 as a control, was used to investigate thymol‐rich essential oil apoptotic activity. The results showed that the 4 T1 tumor line was inhibited without substantially affecting normal L929 cells. The apoptosis was due to ROS production, variations in the mitochondrial membrane, caspase‐3 activation, and DNA damage (Jamali et al. 2020). Thymol derivatives showed better antiproliferative activity than common chemotherapeutic drugs like 5‐fluorouracil against BC cells (MCF‐7 and MDA‐MB‐231). Thymol derivative, known as compound 10 (IC50 6.17 μM) exhibited 3.2‐fold more inhibition than 5‐fluorouracil (IC50 20.09 μM) against MCF‐7 cell lines, while (IC50 10.52 μM) of compound 10 proved 1.42 and 2.4 times better than tamoxifen (IC50 15.01 μM) and 5‐fluorouracil (IC50 25.31 μM) respectively, in MDA‐MB‐231 cell lines (Alam et al. 2021). *Conobea scoparioides* essential oil (EO) *containing* thymol methyl ether (62%) and thymol (16%) proved effective in in vitro and in vivo studies. The 40 and 80 mg/kg EO reduced tumor mass to 36.7% and 55.8%, respectively, whereas in vitro analysis showed that EO induced apoptosis in HepG2, HCT116, and MCF‐7 cell lines (de Lima et al. 2020).

The association between Hedgehog/GLI (HH/GLI) signaling pathway and oncogenesis has been established in studies. This pathway is involved in cell growth and stem cell maintenance, primarily active in the embryonic stage, wound healing, and tissue repair. The irregularities in this pathway result in brain, lung, pancreas, cervical, and prostate cancer development (Trocchianesi et al. 2023). Carvacrol has been reported to have the potential to induce apoptosis and inhibit cell multiplication through modulation of the HH/GLI pathway in C33A cervical cancer (CC) cells. Carvacrol (25, 50, 75, and 90 μM) enhanced the expression of *Bax*, *Bad*, *Fas‐L*, and *cytochrome c*, activated caspase‐9/3 and caspase‐8, induced cell cycle at G0/G1, improved the expression of proteins (p21, cyclin D1, CDK4), and downregulated the SMO and GLI1 proteins expression in CC (Ahmad et al. 2023). The *p53* gene regulates the transcription of *Bcl‐2* and *Bax* genes and modulates *Bax/Bcl‐2* expression. However, microRNA‐21 (miR‐21) and TTDA protein inhibit the expression of *p53*, resulting in alterations in *Bcl‐2*/*Bax* expression (Luo et al. 2020). Carvacrol (305 μM) induced apoptosis in MCF‐7 bc cell lines via modulating p53/Bax/Bcl‐2 axis. The findings demonstrated that carvacrol improved the expression of *p53*/*Bax*, while downregulated *Bcl‐2* (Moradipour et al. 2022). Similarly, carvacrol (0–250 μmol/L) initiates apoptosis and cell cycle arrest against MCF‐7 cancer cell lines by modulating the PI3K/AKT pathway. The results revealed that carvacrol (200 μmol/L) at 24 and 48 h significantly inhibited cell proliferation, triggered apoptosis by inhibiting p‐Rb, CDK4, CDK6, and cyclin D1, decreased *Bcl‐2*, and improved *Bax* expressions (Mari et al. 2021).

### 3.2 Cell Cycle Arrest

Cancer is characterized by dysregulation and abnormality in the cell cycle regulation. The cell cycle is divided into two phases: the interphase and the mitosis (M) phase, involving the division of cell components and conversion of the cell into two identical daughter cells (Matthews et al. 2022). The disrupted cell cycle leads to cell proliferation and cancer, and irregular cell cycle checkpoints increase the risk of genomic instability. Studies on thymol have reported its role in cell cycle arrest at different stages through several mechanisms. Balan et al. (2021) reported that thymol (112 μg/mL) caused cell cycle arrest at the G0/G1 phase and triggered apoptosis in A549 cells. They concluded that upregulation of *Bax*, suppression of *Bcl‐2* expression, increased ROS production, elevated lipid peroxides, and mitochondrial membrane depolarization are substantial mechanisms responsible for cell death. Thymol extracted from *Thymus vulgaris* exhibited in vivo and in vitro anticancer potential against HCT116 and Lovo cell lines. Thymol (75 and 150 mg/kg) reduced tumor volume, induced cell death, and modulated BAX/Bcl‐2 and Wnt/β‐catenin pathways in an in vivo analysis. In vitro study showed that thymol (0, 10, 20, 40, 80, and 120 μg/mL) enhanced apoptosis and induced cell cycle arrest in cell lines (Zeng et al. 2020). Current studies have confirmed that thymol can improve the potential of chemotherapeutic drugs to obstruct cell proliferation and cancer progression. In an in vitro study, the synergetic impact of thymol on 5‐Fluorouracil (5‐FU) against KYSE‐30 esophageal cancer (EC) cells has been reported. Thymol enhanced the cytotoxicity of 5‐FU via augmented ROS generation, improved *p53* and *Bax* expression, decreased *Bcl‐2* expression, and declined MMP‐2 activity. Moreover, combined therapy induced cell cycle arrest at the G2/M phase (Pouyamanesh et al. 2024). Thymol induced cell cycle arrest at the G0/G1 phase, enhanced ROS production, caused apoptosis, and activated caspase‐9/3 in CRC cell lines (Anvarbatcha et al. 2023).

Carvacrol is a less studied monoterpene compared to thymol concerning cell cycle arrest. Previous studies have reported its impact and potential in cell cycle arrest. Notch signaling is closely linked with prostate cancer (PC), and carvacrol has been proven effective in inhibiting PC progression. Carvacrol induced apoptosis and cell cycle arrest at the G0/G1 phase via ROS production, *BAX/Bcl‐2* expression modulation, and cyclin D1 and CDK4 inhibition. In addition, carvacrol modulated Notch signaling in PC by downregulating Notch‐1 and Jagged‐1 expression (Khan et al. 2019). Drug‐resistant cancer cells could be the major problem in cancer treatment. Drug resistance occurs due to molecular changes in cancerous cells; moreover, genetic mutations in cancer cells that affect genes targeted by drugs and genetic modifications in genes that are responsible for repairing DNA damage are other aspects that are leading to drug resistance (Garg et al. 2024). Carvacrol induced apoptosis in doxorubicin‐resistant A549 lung cancer cells, proved by higher expression of Bax, cytochrome c, and caspase 3/9. Moreover, cell cycle arrest was noticed due to reduced CDK2, CDK4, cyclin D1, and enhanced p21 protein expression and declined autophagy marker ATG5 (Khan et al. 2018). In another study, carvacrol induced cell cycle arrest at the G0/G1 phase by ROS production against DU145 PC cell lines, which were reported, confirmed by caspase‐3 activation and mitochondrial membrane potential disruption (Khan et al. 2017).

## 4 Modulation of Signaling Pathways

The MAPK/ERK pathway is a crucial signaling pathway involved in cell division and differentiation. An extracellular stimulus, such as growth factors, binds to receptors on the cell surface, thus triggering the pathway, and dysregulation of this pathway promotes cancer development (Hendrikse et al. 2023). The PI3K/AKT signaling pathway is vital in cell proliferation, and its impairment is commonly observed in several cancers, making it a substantial target for anticancer remedies (Glaviano et al. 2023). Like others, Wnt/β‐catenin, JAK/STAT, and NF‐κB pathways are other significant signaling pathways in cell multiplication, inflammatory response, and cytokine signal transmission (Xue et al. 2023; Zhang et al. 2021).

The studies on thymol's anticancer potential showed that it can modulate all these pathways. Thymol can hinder the phosphorylation of vital components in the pathway (MEK1/2 and ERK1/2), leading to decreased cell multiplication and augmented apoptosis across different cancers. Moreover, thymol also inhibits MPPs and other invasion proteins, thus contributing to the modulation of the MAPK/ERK pathway (Guo et al. 2020). Multiple concentrations of thymol (0, 25, 50, 100, and 150 μM) were evaluated against bladder cancer cell lines (T24 and J82). It was found that thymol induced cell cycle arrest at the G2/M phase via caspase‐3/9 stimulation, downregulating Bcl‐2 family proteins and ROS production. Moreover, thymol activated JNK and p38 MAPK while impeding the ERK pathway, indicating that JNK and p38 are critical factors for apoptosis (Li et al. 2017). Thymol (50, 100 mg/kg) remarkably protected against pulmonary fibrosis by regulating PI3K/AKT signaling in mice via reduced TNF‐α, IL‐6, NF‐kB, and TGF‐β expression (Hussein et al. 2023). Thymol reduced cell proliferation and augmented apoptosis in lung carcinoma by reducing AKT phosphorylation, thus exerting an anticancer effect via PI3K/AKT pathway modulation (Sampaio et al. 2021). Another study reported that thymol decreased HT29 cell migration, inhibited MMP‐2/9 activity, and downregulated PI3K/AKT and ERK pathways (Lv and Chen 2017). Khan et al. (2024) stated that thymol inhibited STAT3 phosphorylation and downregulated JAK/STAT signaling, thus showing antiproliferative properties. The STAT3 is interlinked with HIF‐1α, as both are transcription factors and can cooperate to regulate gene expression, especially in response to hypoxia. STAT3 can increase the stability and activity of HIF‐1α, leading to augmented expression of HIF‐1α target genes and angiogenesis (Dinarello et al. 2023). Thymol (30 mg/kg/day), along with thymoquinone (10 mg/kg/day), alleviated MSG‐induced ADHD in rats via modulating Wnt/β‐Catenin, Nrf2/HO‐1, and TLR4/NF‐κB/NLRP3/caspase‐1 pathways (Abu‐Elfotuh et al. 2022).

**FIGURE 3:**
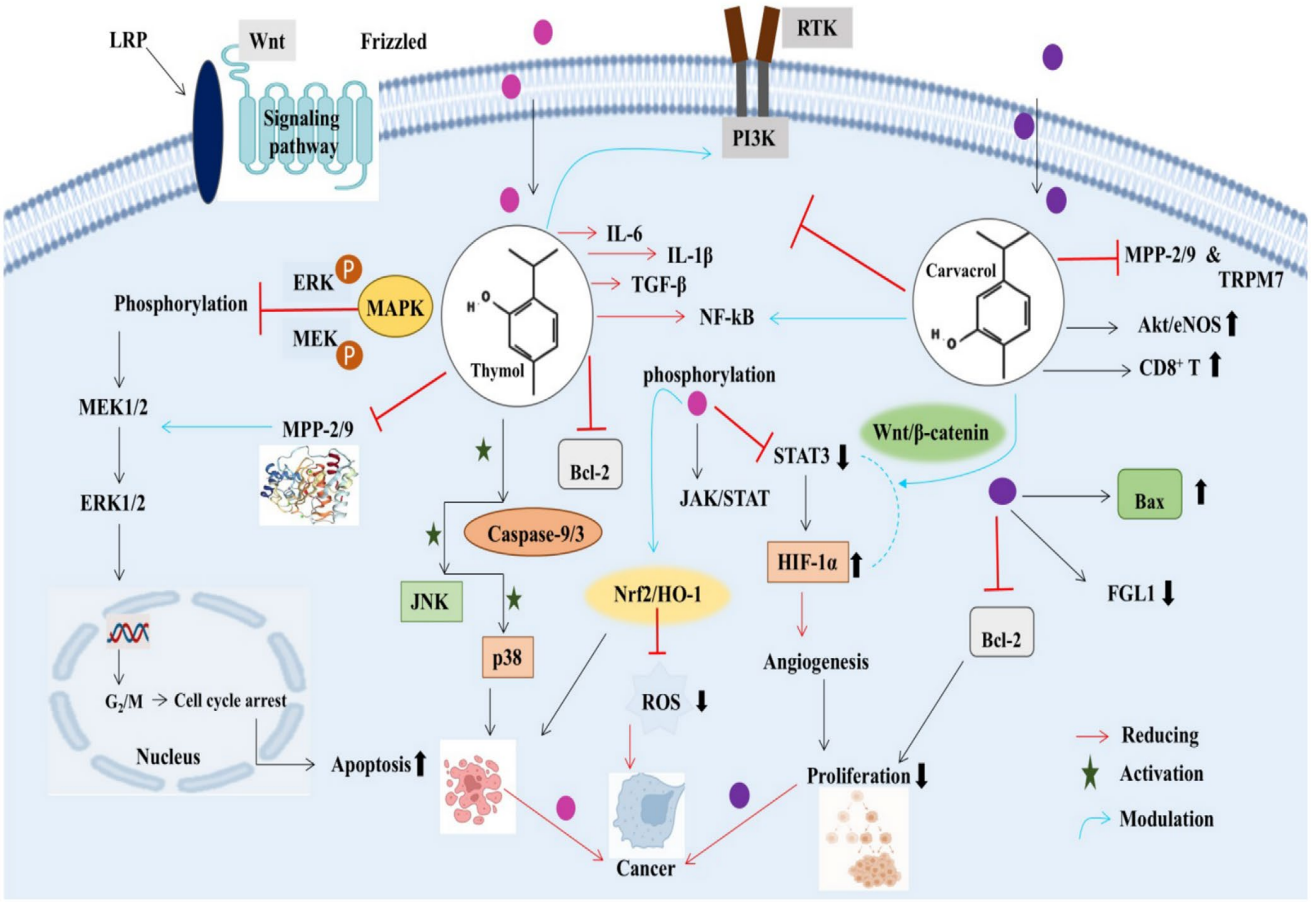
Modulation of MAPK/ERK, PI3K/AKT, Wnt/β‐catenin, and JAK/STAT by thymol and carvacrol via cell cycle arrest, apoptosis induction, inhibited phosphorylation of MEK1/2, ERK1/2, and STAT3, caspase‐3/9, JNK, and p38 MAPK activation, downregulation of Bcl‐2 proteins, reducing TNF‐α, IL‐1β, IL‐6, and TGF‐β expression, regulation of Nrf2/HO‐1, TLR4/NF‐κB/NLRP3/caspase‐1, and HIF‐1α/STAT3/FGL1 pathways, upregulation of AKT/eNOS pathway, and inhibition of MMP‐2 and TRPM7.

Besides thymol, carvacrol has also been reported to inhibit MAPK or ERK pathways in previous studies. Carvacrol (25, 50, and 100 mg/kg) for 6 weeks modulated the MAPK signaling pathway and inhibited TRPM7 expression in liver fibrotic C57BL/6J mice (Cai et al. 2021). Carvacrol (25, 50 and 100 mg/kg) modulated the ERK pathway, upregulated the AKT/eNOS pathway while imposing no impact on p38MAPK and JNK in myocardial ischemia/reperfusion injury, proving its cardioprotective potential (Chen et al. 2017). The PI3K/AKT is connected with TRPM7, a channel kinase that activates the PI3K/AKT pathway. Carvacrol (500 μM) inhibited cell proliferation in PC‐3 and DU145 PC cancer cells by inhibiting MMP‐2 and TRPM7 channels and suppressing PI3K/AKT signaling pathways (Luo et al. 2016). Carvacrol (1, 3, and 10 mg/kg body weight) for 48 h ameliorated cisplatin‐induced renal injury in BALB/cN mice. The study demonstrated that carvacrol alleviated renal injury by modulating p‐NF‐κB, PI3K/AKT, and ERK pathways (Potočnjak and Domitrović 2016). Carvacrol (15 mg/kg) combined with sorafenib modulated HIF‐1α/STAT3/FGL1 pathway in a hepatocellular carcinoma (HCC) murine model. The findings showed that carvacrol increased CD8^+^ T cells and reduced animal fibrinogen‐like protein 1 (FGL1) expression. In a xenograft animal model, carvacrol proved effective against osteosarcoma by regulating the Wnt/β‐catenin pathway. Moreover, the study revealed that carvacrol treatment enhanced *Bax* while decreasing *Bcl‐2* and MMP‐9 expression (Zhang et al. 2022). The modulating impact of thymol and carvacrol on different cancer pathways is illustrated in Figure 3.

## 5 Anticancer Perspective of Thymol and Carvacrol

Cancer is affecting millions of people worldwide; however, numbers are increasing in middle‐ and low‐income countries due to poor and unhygienic dietary choices, polluted environmental conditions, and prevailing pathogenic diseases. Thymol and carvacrol, monoterpenes from essential oils of herbs, exhibit anticancer potential through apoptosis induction, OS reduction, tumor growth inhibition, and modulation of TSGs and oncogenes. The anticancer potential of thymol and carvacrol and possible mechanisms are mentioned in Table 1.

**TABLE 1:** Anticancer studies of thymol and carvacrol through possible mechanisms.

Compound	Cancer	Study	Mechanism/outcome	References
Thymol	Colorectal cancer	In vitro	**↑**Apoptosis, **↓**EGFR genes expression	Keshavarz et al. (2024)
			**↑**ROS, cell cycle arrest at G0/G1 phase, **↑** *p53* expression, **↓** *Bcl‐xL*	Anvarbatcha et al. (2023)
	Breast cancer	Network pharmacology	Modulated *PIK3CA*, **↓**proliferation	Laamari et al. (2025)
		In vitro	**↓**COX‐2 and cell proliferation, **↑**apoptosis	Taibi et al. (2024)
	Lung cancer	In vitro	**↑**ROS, **↑**SphK1	Shakeel et al. (2024)
			**↑**Apoptosis	dos Santos et al. (2021)
	Liver cancer	In vitro	**↓**VEGFR and VEGF genes expression	Hussein (2024)
		Molecular docking	Bind and inhibit P38 Protein	Tabassum and Ahmad (2020)
	Esophageal cancer	In vitro	**↑**ROS, *p53* and *Bax*, **↓** *Bcl‐2* and MMP‐2, cell cycle arrest at G2/M phase	Pouyamanesh et al. (2024)
	Ovarian cancer	In vitro	**↓** *Bcl‐2*, **↑**caspase‐3/8/9 and *Bax*	Seçme and İlhan (2025)
			Apoptosis induction	Elbe, Yigitturk, Cavusoglu, Baygar, et al. (2020)
	Bladder cancer	In vitro	Cell cycle arrest at the G2/M phase, **↓** *Bcl‐2*, caspase‐3/9 activation	Li et al. (2017)
	Prostate cancer	In vitro	Induced apoptosis, **↓**cell viability	Yeh et al. (2017)
			Induced apoptosis, caspase‐3 activation	Singhal et al. (2021)
	Cervical cancer	In vitro	**↑**Apoptosis	Osarieme Imade et al. (2023)
	Skin cancer	In vitro	Modulation of VEGF and VEGFR genes expression	Feyzmohamadi Khoramabadi et al. (2024)
Carvacrol	Colorectal cancer	In vitro	**↓**Cell proliferation and migration	Abed et al. (2024)
	Breast cancer	In vitro	**↓** *Jagged‐1* and *cyclin D1*, modulated NOTCH pathway	Pandey et al. (2024)
	Breast cancer	In vitro and in vivo	Modulated PI3K/AKT/mTOR, ↑OS and PTEN expression	Srinivasan and Namasivayam (2024)
		In vitro	Cell cycle arrest at S and G2/M phase, **↓** *Bcl‐2* and *PI3K* expression	Chen et al. (2024)
			Cell cycle arrest at G2/M phase, apoptosis induction	McClements (2024)

	Lung cancer	In vitro	Cell cycle arrest at G2/M phase and apoptosis induction	Bansal et al. (2022)
		In vitro and molecular docking	Modulated EGFR and BRAF proteins	Çakır et al. (2025)
	Gastric cancer	In vitro	**↓** *Bcl‐2*, **↑** *Bax*	Elahi et al. (2022)
	Prostate cancer	In vitro	**↑**JNK and p53 proteins	Razack et al. (2025)
	Cervical cancer	In vitro	Cell cycle arrest at G0/G_1_ phase, **↓** *E6* and *E7* oncogenes, **↑** *p53*expression	Ahmad et al. (2025)
			**↓**Cell proliferation	Akhlaq et al. (2023)
	Liver cancer	In vitro	Improved Topotecan cytotoxicity	Bayoumi et al. (2021)
	Pancreatic Cancer	In vitro	Induced apoptosis, **↓**CDH1, TIMP1, TIMP3, and ZEB1expression	Gunes et al. (2022)
	Plasma cell cancer (Myeloma)	In vitro	**↓**MMP level, *Bcl‐2*, **↑** *Bax*, caspase‐3/9, ROS, apoptosis	Zhang et al. (2023)
Skin cancer	In vitro	Induced apoptosis, **↓**metastasis	Osanloo et al. (2023)

### 5.1 Breast Cancer

Breast cancer (BC) is the 2nd leading cancer among different cancers and ranked 1st among women concerning prevalence worldwide, and according to WHO, ~2.3 million women were identified with BC in 2022 (Nardin et al. 2020). Hormonal replacement therapy (HRT) is a key risk factor for BC. However, risk can be limited if HRT is used after 60 years (Smolarz et al. 2022). Genetic mutations are only counted in 5 to 10% of cases of BC development, and *BRCA1* and *BRCA2* are TSGs positioned on chromosomes 17 and 13, respectively, involved in genomic stability, encrypting nuclear protein, and repairing double DNA strand breaks. Some other genes like *CHEK2*, *ATM*, *PALB2*, and *BRIP1* show a modest tendency to BC. However, patients with these gene mutations have 2 to 3 times greater risk of developing malignant tumors (Chamseddine et al. 2022).

Phosphatidylinositol 3‐kinase (PI3K) is a key factor responsible for cell growth, proliferation, and motility and works with mTOR and AKT to regulate all these cellular mechanisms. The dysregulation of this PI3K/AKT/mTOR pathway is involved in multiple cancer developments, including BC (Bertucci et al. 2023). However, thymol derivative 1,2,3‐triazoles have been proven to be a potent anticancer agent. In this context, Laamari et al. (2025) investigated 1,2,3‐triazoles against breast cancer by targeting *PIK3CA* and concluded that 1,2,3‐triazoles modulate *PIK3CA* to regulate cell growth and proliferation. Thymol (0–1000 μM) concentrations from *N. sativa* were evaluated against MCF‐7 bc cells, and it has been found that thymol substantially inhibited 50% of cell growth at 200 μM concentration. Moreover, it downregulated *cyclin D1* and *PCNA* gene expression to inhibit cell invasion (Vahitha et al. 2024). Recent novel strategies such as nanoparticles (NPs) have emerged as promising anticancer therapies due to their effectiveness in releasing drugs/compounds at the targeted locations. Thymol‐iron oxide NPs were applied against MCF‐7 cancer cell lines to study their cytotoxic impact on cancer cells. The study showed that 90.4 μg/mL of NPs effectively inhibited 50% of cell growth and improved the *BAX* and *CASP8* gene expression (Fekri Kohan et al. 2023). Similarly, Alam et al. (2021) examined thymol 1,2,3‐triazole derivatives against MCF‐7 cell lines. They concluded that 1,2,3‐triazole (IC50 6.17 μM) proved more potent against MCF‐7 cells compared with 5‐FU (IC50 20.09 μM). Furthermore, compound 10 showed (IC50 10.52 μM) against MDA‐MB‐231 cells compared with 5‐FU (IC50 25.31 μM) and tamoxifen (IC50 15.01 μM). Consequently, thymol derivatives induced cell cycle arrest at the G2/M phase via inhibiting thymidylate synthase.

**FIGURE 4:**
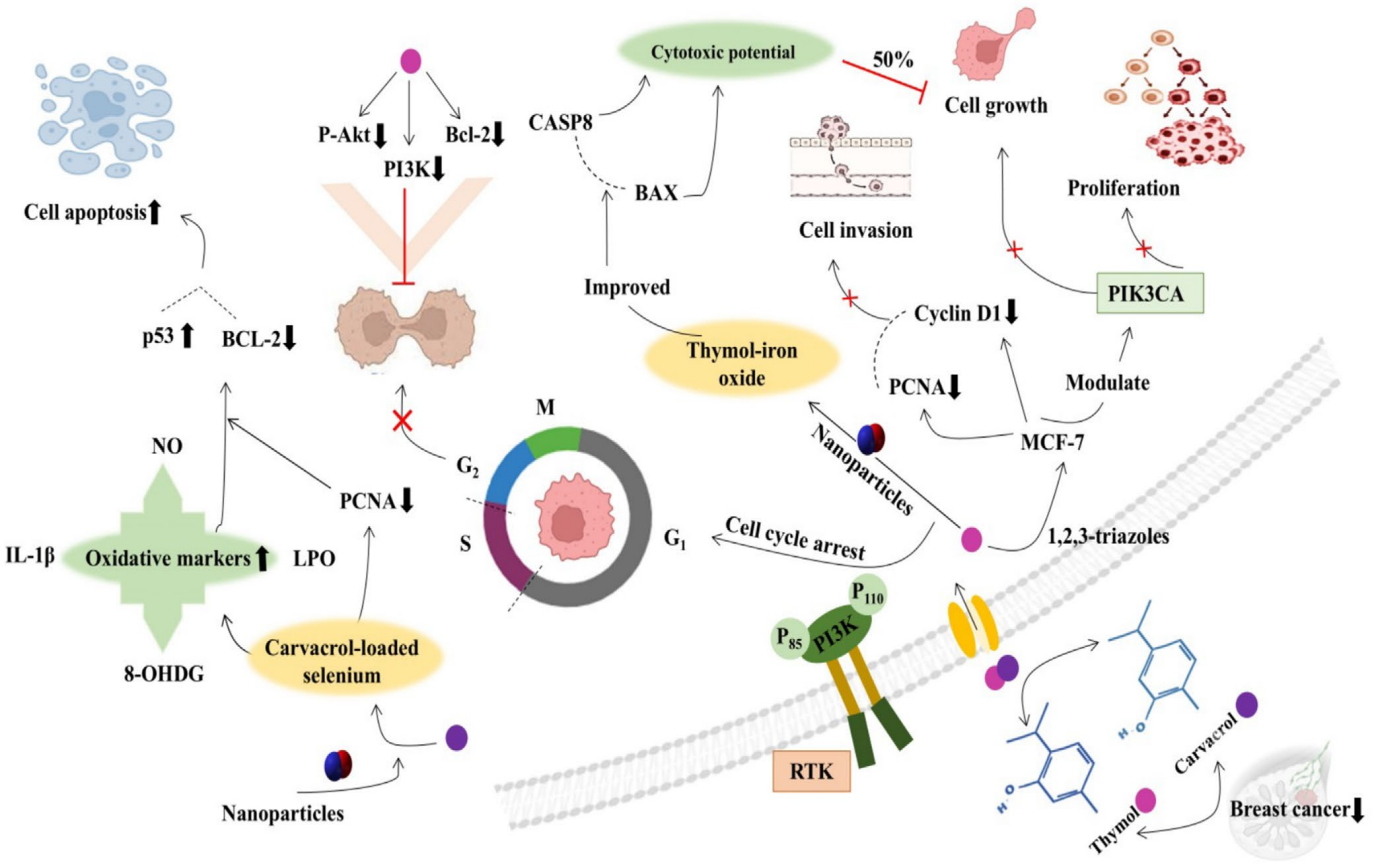
Anticancer role of thymol and carvacrol in breast cancer via cell cycle arrest, apoptosis induction, oxidative stress production, enhancing NO, LPO, IL‐1β, and 8‐OHDG, downregulating *PCNA*, *cyclin D1*, *BCL‐2*, and *PI3K*, upregulating *p53*, *BAX*, and CASP8 expression, and modulating *PI3KCA*.

Along with thymol, carvacrol has been reported to have remarkable potential to ameliorate BC. Carvacrol (0–65 μM) significantly reduced MDA‐MB‐231 cell proliferation via downregulated *Jagged‐1* and *cyclin D1* gene expression and modulation of the NOTCH pathway (Pandey et al. 2024). Chemotherapeutic drug resistance could be a major obstacle to attenuating cancer progression. Chen et al. (2024) reported that carvacrol (0, 100, 150, and 200 μM) inhibited cell development and induced apoptosis in Doxorubicin (Dox) resistant MDA‐MB‐231 breast cancer cells via cell cycle arrest at S and G2/M phases, enhanced *Bax* expression, while reduced *Bcl‐2*, *PI3K*, and *P‐AKT* expression. Carvacrol‐loaded selenium NPs (8.3 μg/mL) proved effective in inducing apoptosis in MCF‐7 bc cell lines by increasing OS markers (8‐OHDG, IL‐1β, NO, and LPO) and downregulated *PCNA* expression (Othman et al. 2023). Moradipour et al. (2022) stated that carvacrol (305 μM) inhibited MCF‐7 cell lines via enhancing *Bax* expression, suppressing *Bcl‐2* expression, and induced apoptosis through upregulation of *p53* expression. The anticancer mechanism of thymol and carvacrol in breast cancer is illuminated in Figure 4.

### 5.2 Lung Cancer

Lung cancer (LC) is the leading cancer among all cancers, including small cell lung cancer (SCLC) and non‐small cell lung cancer (NSCLC). Several factors are involved in LC, and smoking is the leading risk factor responsible for almost 85% of cases. Smoking can lead to dysplasia of lung epithelium, which may cause genetic alterations and affect protein synthesis upon continuing. Genetic mutation in *MYC*, *Bcl‐2*, and *p53* genes is responsible for causing NSCLC (Kenzerki et al. 2023). Moreover, the dysfunction of the RB family (p107, p130), tumor suppressor *PTEN*, chromatin regulator CREBBP, and NOTCH receptors are other factors contributing to cancer pathogenesis. PI3K/AKT/mTOR pathway activation is also related to SCLC progression (Lázaro et al. 2022).

Sphingosine‐1‐phosphate (S1P), activated through catalytic actions of sphingosine kinase 1 (SphK1), is a key factor responsible for cell growth, survival, migration, and angiogenesis and is involved in neoplasm (Bravo et al. 2022). A comparative study of thymol and thymoquinone used as SphK1 inhibitors against NSCLC H1299 and A549 cell lines showed that both compounds remarkably inhibited SphK1 with the IC50 values of 35.52 μM and 53.68 μM, respectively. However, thymoquinone exhibited a better cytotoxic effect than thymol, and the IC50 values were 27.96 μM for H1299 and 54.43 μM for A549 lines (Shakeel et al. 2024). Thymol (80, 120, and 180 μM) extracted from *Thymbra spicata* was evaluated against the NSCLC cell line A549, and it was found that thymol induced a cytotoxic effect on the cell line via activation of NOX2, ROS production, increased Cai2^+^, and *Bax/Bcl‐2* ratio (Moayeri et al. 2021). Similarly, carvacrol from ethanolic *Origanum vulgare* extract showed cytotoxic activity against A549 LC cell lines. The results revealed that carvacrol was the major compound in the extract, and the cellular uptake of carvacrol by cells was 56 μM. Moreover, (0–250 μg/mL) *Origanum vulgare* ethanolic extract reduced cell proliferation and induced cytotoxicity (Coccimiglio et al. 2016). The AXL receptor tyrosine kinase (RTK) is linked with cell proliferation, migration, and neoplasm and also promotes epithelial‐mesenchymal transition (EMT), a significant factor in drug‐resistance metastasis (Yan et al. 2021). Carvacrol has been reported to reduce AXL overexpression and suppress cell propagation and migration in NSCLC (Jung et al. 2018).

### 5.3 Colorectal Cancer

Colorectal cancer (CRC) is the 3rd leading cancer and 2nd in casualties globally. CRC develops from the growth of tiny cell groups (polyps) inside the colon, and somehow with age, these polyps transform into tumors in the next 5–10 years. Inherited disorders, genetic alterations, inflammatory bowel disease (IBD), and other malignancies are risk factors for CRC progression (Al‐Muswie et al. 2023). The epigenetics and genetics of CRC involve microRNAs (miRNAs) that induce cancer‐associated pathway disturbances at the post‐transcriptional level. The disturbances result in alterations in oncogenes and TSGs, metastasis, and dysplastic epithelium in the adenoma‐carcinoma process, eventually leading to CRC development (Fischer et al. 2019).

Numerous studies have discussed the association between growth factors and neoplasm. Epidermal growth factor receptor (EGFR) is a tyrosine kinase receptor that plays a significant role in CRC by stimulating several pathways like RAF, MAPK, RAS, and MEK. EGF receptor inhibition has been reported as a practical approach to treating CRC (Janani et al. 2022). Keshavarz et al. (2024) investigated the anti‐EGFR activity of thymol‐based nanoliposome in SW84 and SW111 CRC cell lines and found that thymol‐nanoliposome (IC50 14.2 and 6.4 μg/mL) inhibited SW48 and SW1116 cells growth respectively. Moreover, a significant reduction in EGFR gene expression and enhanced apoptosis was noticed. Thymol (100–1000 μM) showed an antiproliferative effect against HCT‐8 CRC cell lines via cell cycle arrest at G0/G1 phase, upregulating pro‐apoptotic *p53* expression, suppressing anti‐apoptotic *Bcl‐xL* expression, activating caspase‐9/3, and OS‐induced apoptosis (Anvarbatcha et al. 2023). Thymol derivatives with admirable anticancer properties have been studied against HT‐29 and HCT‐116 cell lines. The findings of the study demonstrated that acetic acid thymol ester (IC50 0.08 μg/mL) increased ROS production, induced apoptosis, and subdued cell proliferation in cancerous cells, compared with standard thymol (IC50 ~ 60 μg/mL) (Blažíčková et al. 2022). The activation of Wnt/β‐catenin signaling is noticed in 90% of CRC cases, thus playing a main role in CRC progression. The mechanism is a series of complex steps involving WNT‐protein ligands binding with LRP5/6 membrane receptors, translocation of β‐catenin, and activation of *c‐Myc* and *cyclin D1*, subsequently leading to cell proliferation, migration, and oncogenesis (He and Gan 2023). Previously, Zeng et al. (2020) reported that thymol (75 and 150 mg/kg) from *T. vulgaris* reduced tumor volume and cell metastasis by activating the Bax/Bcl‐2 pathway, suppressing EMT, and regulating the Wnt/β‐catenin pathway.

**FIGURE 5:**
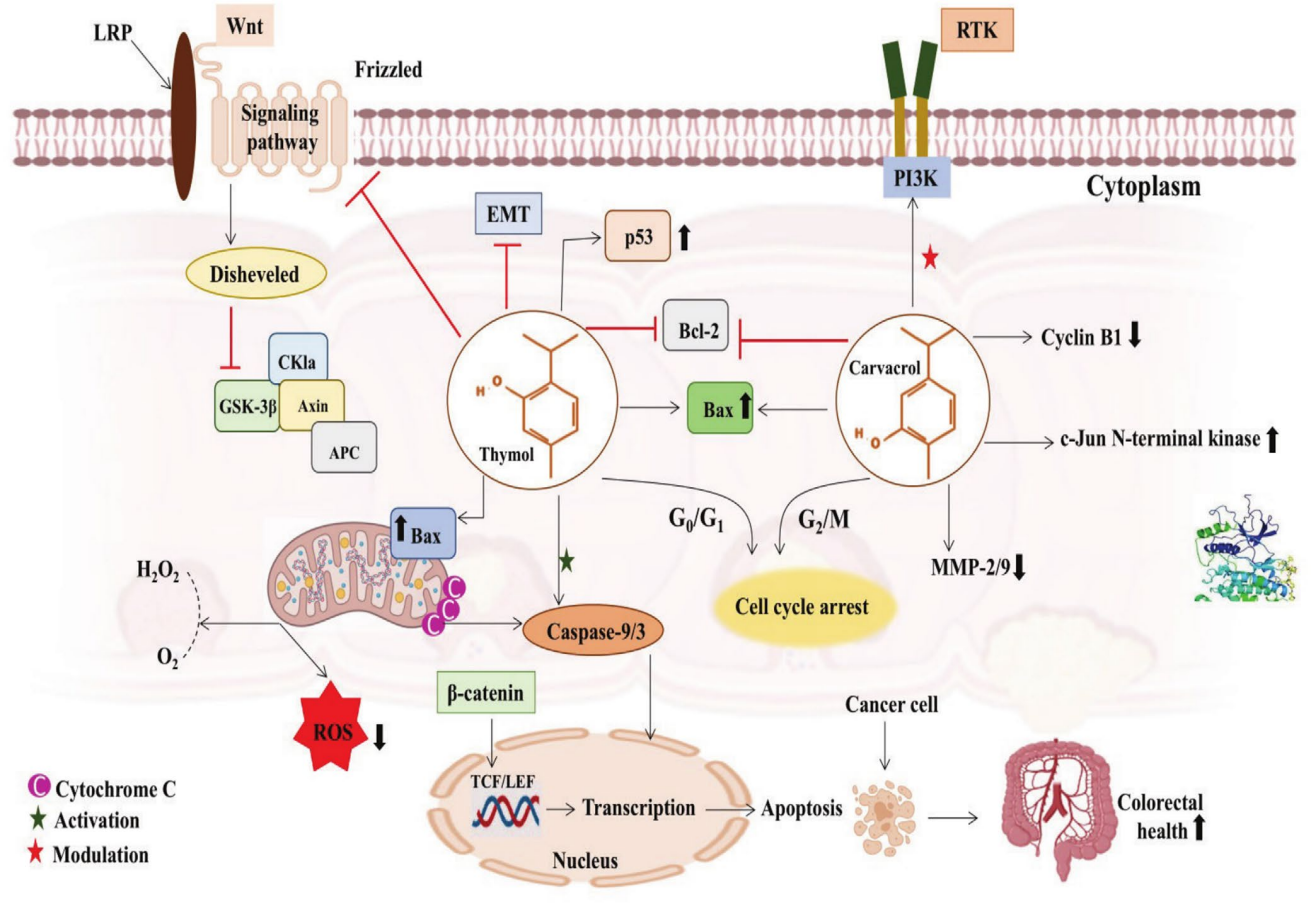
Anticancer mechanism of thymol and carvacrol against CRC via cell cycle arrest, apoptosis induction, Wnt/β‐catenin, HIF‐1α/VEGF, and PI3K/AKT pathways modulation, downregulating MMP‐2/9, *Bcl‐2*, *cyclin B1*, EGFR, and upregulating *Bax*, *p53*, and *c‐Jun N‐*terminal kinase expression.

The studies have established that VEGF promotes matrix metalloproteinases (MMPs) expression, degrades the extracellular matrix, and mediates HIF‐1α. Thus, HIF‐1α contributes significantly to cell migration, proliferation, and induced EMT in malignant tissue. Several chemotherapeutic drugs have been applied to modulate the HIF‐1α/VEGF signaling pathway, but their hostile outcomes, like anemia and appetite loss, are alarming (Chen et al. 2022). Carvacrol has been investigated in a chemical‐induced hypoxic CRC cell model. It was found that carvacrol (6.25, 12.5, 25, and 50 μg/mL) inhibited cell migration in SW480 cell lines. Moreover, carvacrol at higher concentrations suppressed the HIF‐1α/VEGF pathway to inhibit cell proliferation. Another study showed that carvacrol reduced HCT116 and LoVo cell lines invasion via cell cycle arrest at the G2/M phase, diminishing MMP‐2/9, *Bcl‐2*, and *cyclin B1* expression, upregulating *Bax* and *c‐Jun N*‐terminal kinase expression, and modulating MAPK and PI3K/AKT signaling pathways (Fan et al. 2015). Figure 5 demonstrates the anticancer mechanism of thymol and carvacrol against CRC.

### 5.4 Liver Cancer

Liver cancer or hepatocellular carcinoma (HCC) is the 6th most common cancer, affecting approximately 41,630 people globally. Liver cirrhosis, fatty liver disease (FLD), alcohol consumption, exposure to toxins and chemicals, hepatitis, and congenital disorders are major risk factors contributing to HCC development (Huang et al. 2021). Viral hepatitis, a significant risk factor of HCC, mainly alters genes such as *TERT*, *PDGFR β*, and *MAPK1*. Moreover, the virus alters other proteins like HBx, which can mutate *Ras*, *JNK*, *Raf*, *MAPK*, and *ERK* gene expression (Wang and Deng 2023).

Studies have shown that VEGF is often overexpressed in HCC compared to normal liver tissue, especially VEGF‐A, which is the isoform responsible for angiogenesis and vascular remodeling, and its receptors (VEGFRs) are critical for HCC growth and development (Jia et al. 2021). Moreover, hypoxia is a key player in VEGF activation, angiogenesis, and carcinogenesis. In this regard, thymol nanoemulsions have been proven effective against liver cancer via modulating VEGF gene expression (Hussein 2024). Thymol (11 μM) induced apoptosis and genotoxicity in HepG2 cell lines but did not alter the expressions of *Bax, Bcl‐2*, and caspase‐3. Furthermore, several studies on thymol's protective effect against liver injury via regulating inflammatory markers and pathways have demonstrated that it has the potential to inhibit inflammation and genetic modifications, eventually protecting from tumor growth and cell invasion (Yousef et al. 2023). Molecular docking of thymoquinone and thymol from *Nigella sativa* L. inhibited P38 protein, showing that it could be the probable remedy for HCC (Tabassum and Ahmad 2020).

Studies on carvacrol also proved its potential to induce apoptosis in cancer cells and modulate signaling pathways to reduce cell proliferation. Carvacrol (15 mg/kg) by intragastric tube for 6 weeks daily reduced HIF‐1α and downregulated STAT3, JAK2, and FGL1, thus inhibiting cell division and improving immunity via enhancing CD8^+^ T cells (Yousef et al. 2024). Carvacrol (15 mg/kg/day) enhanced antitumor activity combined with sorafenib (10 mg/KG/day) orally in the HCC rats' model. The findings showed that combined therapy induced apoptosis in cancer cells by reducing cyclin D1 *and Bcl‐2* expression while improving *Bax* and caspase‐3 expression. Additionally, the downregulation of ABCG2, NOTCH1, SALL4, and CD133 was observed in the study results (Yousef et al. 2023). An in vitro analysis unveiled that carvacrol (45 μg/mL) and essential oil (0.08 μg/mL) of *Origanum onites* altered the expression of 48 genes out of 84 in HepG2 cells. These 48 genes are associated with HCC and inflammatory pathways, thus proving them a promising anticancer and hepatoprotective agent (Tomsuk et al. 2024). Previously, Yin et al. (2022) reported that carvacrol inhibited cell proliferation and invasion in HepG2 cell lines by targeting 40 genes. They concluded that carvacrol affected *SLC6A3* and *SCN4A*, and the downregulation of *SLC6A3* inhibited cell viability.

### 5.5 Blood Cancer

Blood cancer is a generic term encompassing different types of cancer, like leukemia, lymphoma, and myeloma. Leukemia is one of the most prevalent blood cancers, affecting 474,519 people in 2020, thus making it the 12th most common cancer worldwide (Huang et al. 2022). Acute myeloid leukemia (AML) is the most common type of leukemia, featured by the overproduction of abnormal blood cells, specifically immature white blood cells called myeloblasts. It mainly affects the blood and bone marrow (Wachter and Pikman 2024).

An in vitro study conducted on human AML cell lines (K562, KG1, and HL60) showed that both thymol (25, 50, 75, and 100 μM) and carvacrol (100, 200, 300, and 400 μM) induced caspase‐dependent apoptosis in HL60, while inducing caspase‐independent apoptosis in KG1 and K562 (Bouhtit et al. 2021). Studies on terpenes have proved their effectiveness against leukemia by targeting various factors. *Cymbopogon flexuosus* volatile oil (CFVO) containing terpenes, including thymol and carvacrol, suppressed NF‐κB‐dependent proteins like anti‐apoptotic (cFLIP, Bcl‐xL and XIAP) proliferative (Cyclin E1, Cyclin D) and invasion (ICAM‐1) proteins. Moreover, CFVO also reduced TNF‐α, thus establishing its anti‐leukemic potential against KBM‐5 cell lines (Sajid et al. 2025). Another study reported that thymol‐induced apoptosis modulates signaling cascades to inhibit cell invasion in leukemia cell lines (Logesh et al. 2024).

## 6 In Vitro and In Vivo Anticancer Activity of Carvacrol

Carvacrol exhibits significant anticancer properties in both in vitro and in vivo studies, demonstrating apoptosis induction, cell cycle arrest, and antiproliferative effects against various cancer types, highlighting its therapeutic potential. Carvacrol (25–200 μM) inhibited CRC by reducing HT‐29 cell line proliferation via modulating CDK4, *Cyclin D1*, *Bax*, *and Bcl‐2* expression (Pakdemirli et al. 2020). Likewise, carvacrol (25–500 μM) inhibited MCF‐7 bc cell lines by enhancing *p53*, *Bax*, caspase 3/6/7 expression, downregulating *Bcl‐2* expression, and modulating the PI3K/p‐AKT pathway (Jamali et al. 2018). The SiHa cell lines of cervical cancer revealed apoptosis via increased expression of *Bax* and diminished expression of *Bcl‐2* upon being treated with carvacrol (25–500 μM) (Al‐Fatlawi 2018). Carvacrol (200–1000 μM) modulated caspase‐3 and *Bcl‐2* expression in K562 leukemia cell lines (Bouhtit et al. 2021). The anticancer activity of carvacrol against different cancerous cell lines and in the in vivo model is listed in Table 2.

**TABLE 2:** In vitro and in vivo anticancer potential of carvacrol.

Cancer	Cell lines/animal type	Concentration/dose/route/duration	Molecular targets	References
Breast cancer	MDA‐MB‐231	1–10,000 μM	Cyt c, *Bax*, CDK4	Baranauskaite et al. (2017)
	MCF‐7	25–500 μM	*p53*, *Bax*, caspase 3/6/7, *Bcl‐2* PI3K/p‐AKT	Jamali et al. (2018); Al‐Fatlawi and Ahmad (2014)
	BT‐483, BT‐474	25–500 μM	*cyclin B*, CDK4	Li et al. (2021)
Cervical cancer	HeLa	25–800 μM	*Bax*, *Bcl‐2*, *p53*, caspase‐3/6/9	Mehdi et al. (2011)
	SiHa	25–500 μM	*Bax*, *Bcl‐2*, *p53*, caspase‐3/6/9	Al‐Fatlawi (2018)
Choriocarcinoma	JAR, JEG3	50–300 μM	PI3K/AKT, p‐ERK1/2 p‐p38, MMP	Lim et al. (2019)
Lung cancer	A549	100–1000 μM	*p38*, NF‐κB, AXL, TNFα, GSK‐3b, Beclin1	Jung et al. (2018)
	H460	30–300 μM	AXL	Coccimiglio et al. (2016)
	H1299	25–1800 μM	Cell membrane and DNA damage	Ozkan and Erdogan (2012)
Colorectal cancer	HT‐29	25–200 μM	CDK4, *Cyclin D1*, *Bax*, *Bcl‐2*	Pakdemirli et al. (2020)
	Caco‐2	100–2500 μM	DNA damage	Llana‐Ruiz‐Cabello et al. (2014)
	LoVo	100–900 μmol/L	*Bcl‐2*, *Bax*, MMP‐2/9, *Cyclin B1*	Fan et al. (2015)
	HCT 116	100–900 μmol/L	PI3K/p‐AKT	Fan et al. (2015)
PROState cancer	DU145	10–500 μM	*Bax*, *Bcl‐2*, Notch1, Jagged‐1, MMP2	Tayarani‐Najaran et al. (2019)
	PC‐3	25–800 μM	PI3K/AKT, TRPM7, p‐STAT3, p‐ERK1/2	Luo et al. (2016)
Melanoma	A375	3.906–1000 μg/mL	*Bcl‐2*, cell cycle arrest	Govindaraju and Arulselvi (2018)
Gastric adenocarcinoma	AGS	100–600 μM	*Bax*, *Bcl‐2*, Caspase‐3/9	Günes‐Bayir et al. (2018)
Glioblastoma	U87	1–10,000 μM	PI3K/AKT, MAPK, TRPM7	Chen et al. (2015)
	DBTRG‐05MG	200–1000 μM	Caspase‐3, ROS production	Liang and Lu (2012)
Neuroblastoma	SH‐SY5Y	12.5‐50 μM	*Bax*, *Bcl‐2*	Çalibaşi Koçal and Pakdemirli (2020)
	N2a	10–400 mg/L	TNF‐α, *MYCN*	Aydın et al. (2014)
Oral cancer	Tca‐8113	10–80 μM	CCND1 CDK4, *p21*, *Bcl‐2*/*Bax*, COX‐2	Dai et al. (2016)
	SCC‐25	167 μg/mL	MMP‐2/9 COX‐2, *p21*	Dai et al. (2016)
	OC2 cells	200–1000 μM	Caspase‐3, ROS generation	Liang et al. (2013)
Leukemia	HL‐60	10–400 μM	MMP, *Bcl‐2*	Bhakkiyalakshmi et al. (2016)
	K562	200–1000 μM	Caspase‐3, *Bcl‐2*	Bouhtit et al. (2021)
	KG1	100–400 μM	*pp38*	Bouhtit et al. (2021)
	CEM	0.05–1.25 μM	Cell cycle interruption	Jaafari et al. (2012)
	P‐815	0.05–1.25 μM	Cell cycle arrest	Jaafari et al. (2012)
Liver cancer	Hep3B	1–1000 μM	*Bcl‐2*, *pp38*	Fitsiou et al. (2016)
	HepG2	100–1000 μM	MAPK p‐ERK 1/2, Caspase‐3	Elshafie et al. (2017)
Ovarian cancer	SKOV‐3	100–600 μM	GSK‐3β, NF‐κB	Elbe, Yigitturk, Cavusoglu, Uyanikgil, and Ozturk (2020)
Breast cancer	Holtzman mice	50, 100, 200 mg/kg for 14 weeks (orally)	Antioxidant activity	Rojas‐Armas et al. (2020)
Colon cancer	Wistar rats	20, 40, 80 mg/kg/day for 16 weeks (i.p.)	**↑**GPx, GSH, SOD, CAT	Sivaranjani et al. (2016)
Liver Cancer	Wistar rats	15 mg/kg for 16 weeks (orally)	Improved AST, ALT, ALP, cGT	Jayakumar et al. (2012)
		15 mg/kg for 15 weeks (orally)	**↓**AFP, VEGF, AFU, and GGT PARP	Ahmed et al. (2013)
		15 mg/kg carvacrol for 16 weeks (orally)	**↓**MMP‐2/9, AgNORs, *PCNA*	Subramaniyan et al. (2014)
	C57BL/6 mice	20 weeks (i.g.)	Modulation of DAPK1 and PPP2R2A	Li et al. (2019)

## 7 In Vitro and In Vivo Anticancer Studies of Thymol

In vitro and in vivo anticancer studies of thymol show its potential in inhibiting cancer cell growth, inducing apoptosis, and reducing tumor size, highlighting its promise as a natural anticancer agent. Qoorchi Moheb Seraj et al. (2022) reported that thymol (230 μM) reduced *AKT* and enhanced *p38* expression in U‐87 glioblastoma cell lines, thus effectively decreasing oncogenesis. In another study, 46.74 μg/mL and 41.46 μg/mL thymol inhibited HCT116 and LoVo cell line invasion via downregulating the Wnt/β‐catenin pathway and reducing *c‐Myc* and *Cyclin D1* expression (Zeng et al. 2020). Thymol (15.6 to 2000 μM) induced apoptosis in KYSE‐30 esophageal cancer cell lines through improved ROS production, enhanced *p53*, and *Bax* expression, and declined *Bcl‐2* expression (Pouyamanesh et al. 2024). Thymol (4.3 mM) increased ROS production and induced apoptosis against oral cancer in athymic nude mice (Zeng et al. 2020). The in vitro and in vivo studies of thymol against various cancers are highlighted in Table 3.

**TABLE 3:** In vitro and in vivo anticancer studies of thymol.

Cancer	Cell lines/animal type	Concentration/dose	Molecular targets	References
Glioblastoma	U‐87	230 μM	**↓** *AKT ↑p38*	Qoorchi Moheb Seraj et al. (2022)
Colorectal cancer	HCT116	46.74 μg/mL	**↓**Wnt/β‐catenin	Zeng et al. (2020)
	LoVo	41.46 μg/mL	c‐Myc, Cyclin D1	
Prostate cancer	PC‐3, DU145	100, 200, 400, 600, 800 μM	*Bax*, *Bcl‐2*	Elbe, Yigitturk, Cavusoglu, Uyanikgil, and Ozturk (2020)
Breast cancer	MDA‐MB‐231			
Lung cancer	KLN205			
Colon cancer	HT29	4 mM	PI3K/AKT, **↓** *ERK*	Lv and Chen (2017)
Leukemia	KG1	100 mM	**↑**Apoptosis, OS, autophagy	Bouhtit et al. (2021)
	HL60			
	K562			
Esophageal cancer	KYSE‐30	15.6 to 2000 μM	**↑**ROS production, *p53*, *Bax*, ↓*Bcl‐2*	Pouyamanesh et al. (2024)
Ovarian cancer	OVCAR‐3	145.683 μg/mL and 388.53 μg/mL	**↓** *Bcl‐2*, **↑**caspase‐3/8/9 and *Bax*	Seçme and İlhan (2025)
Prostate cancer	LNCaP	50, 100, 150, 200, and 250 μM	**↑**Apoptosis	Singhal et al. (2021)
Cervical cancer	HeLa	10–1000 μg/mL	**↑**Apoptosis	Osarieme Imade et al. (2023)
Skin cancer	A2058	62.5–500 μg/mL	VEGF and VEGFR genes	Feyzmohamadi Khoramabadi et al. (2024)
Colon cancer	Wistar rats	20 mg/kg/day	↓NF‐κB, ↑Caspase‐3	Hassan et al. (2021)
Oral cancer	Athymic nude mice	4.3 mM	ROS production and apoptosis	De La Chapa et al. (2018)
Colorectal cancer	BALB/c nude mice	75–150 mg/kg once/day	Wnt/β‐catenin	Zeng et al. (2020)
Breast cancer	BALB/c mice, Sprague–Dawley rats	0.1% and 1%	*Bax*, CD44, ALDH1A1, and VEGFR‐2	Kubatka et al. (2019)

## 8 Anticancer Synergistic Role With Other Phytochemicals, Chemotherapy, and Radiotherapy

Several studies have proved that thymol and carvacrol enhance anticancer effects by synergizing with bioactive compounds, chemotherapy, and radiotherapy, improving efficacy, inducing apoptosis, oxidative stress, and modulating inflammation in cancer treatment. Bouhtit and colleagues reported that thymol and carvacrol found in the essential oil of *Ptychotis verticillate* induced cell apoptosis in KG1, HL60, and K562 leukemia cell lines (Bouhtit et al. 2021). The combined therapy of *Zataria multiflora* extract (ZME) comprising thymol, carvacrol, and doxorubicin proved efficacious against lymphoblastic leukemia (ALL) Nalm‐6 cells and induced apoptosis via suppressing anti‐apoptotic proteins (Lashkari et al. 2022). Thymol enhanced the cytotoxicity and antiproliferative activity of 5‐FU against BC and CRC cell lines. In addition, thymol attenuated 5‐FU‐induced intestinal mucositis in the murine model (Badr et al. 2022). Another investigation reported that thymol protected and ameliorated 5‐FU‐induced intestinal mucositis via suppressing the TGF‐β/p38/p‐JNK signaling and inhibiting NF‐κB and TNF‐α (Al‐Khrashi et al. 2022).

The chitosan nanoparticles of thymol oil extract and bee pollen were examined against HepG2 and MCF‐7 cancer cells. The findings showed that the combination promoted apoptosis, disrupted the cell cycle via improved caspase‐3 expression, and downregulated caspase‐9 and *P53* (Alshehri and Abdella 2023). Thymol and carvacrol in nanoemulsions and nanocapsules of *O. glandulosum* Desf. oil exhibited cytotoxic effects on Hep G‐2 and THLE2 cell lines. It was observed that nanocapsules (54.93 μg/mL) revealed better anticancer potential compared with nanoemulsions (131.6 μg/mL) and standard drug 5‐FU (5–400 μg/mL) (Ali et al. 2020). Carvacrol, thymol, γ‐terpinene, methyl carvacrol ether, and p‐cymene are the key components of *Zataria multiflora* essential oil (ZEO) (Torabiardekani et al. 2023). The combination of ZEO and doxorubicin was investigated in a study against PC3 cell lines, and it was found that ZEO enhanced doxorubicin anticancer activity by ROS production and induced apoptosis in PC3 cell lines, showing the synergic role of thymol and carvacrol with chemotherapy (Zare et al. 2021).

Radiotherapy is a vital treatment in the fight against neoplasm, as it exploits DNA damage to eradicate tumors efficiently. However, this treatment can harm healthy cells (Barazzuol et al. 2020). In this context, carvacrol and thymol were evaluated to observe the protective effect on rat ovaries against the side effects of radiotherapy. The study elaborated that both compounds improved ovarian follicle development and recovered hormone levels in ovaries. Furthermore, these compounds counteracted OS, improved IGF‐1 levels, and reduced TNF‐α production in signaling pathways (Mahran et al. 2019). Thymol, along with hesperidin, prevented submandibular gland damage caused by radiation therapy. Both compounds decreased the injury by lessening oxidants and improving levels of antioxidant enzymes; however, thymol displayed better protective activity than hesperidin, indicating its ability to reduce the damage caused by radiotherapy (Sakat et al. 2023). Similarly, chemotherapy also imposes an adverse impact on healthy tissues and organs. The studies on carvacrol have proved that it alleviated cisplatin and doxorubicin‐induced cardiotoxicity in rats via apoptosis induction and modulating PI3K/AKT, Notch/Hes1 pathways (Akaras et al. 2024; Retnosari et al. 2024).

## 9 Carvacrol‐ and Thymol‐Based Hybrids With Anticancer Activity

Carvacrol‐ and thymol‐based hybrid compounds with anticancer potential have opened new avenues in drug delivery systems. Their biocompatibility and promising therapeutic strategies have garnered significant attention in research. Demirbolat et al. (2022) developed carvacrol hybrid molecules and validated their cytotoxic impact on PC‐3, SH‐SY5Y, MCF‐7, NIH/3 T3, K562, and A549 cell lines. The study showed compound 3 with a benzene ring exhibited promising cytotoxic activity against MCF‐7 compared with doxorubicin, with the IC50 of 12.8 μM and 49.05 μM, respectively. However, the thymol hybrid derivative (its isomeric partner) showed an IC50 of 5.96 μM against PC‐3 lines. Another study showed that p‐methoxy thymol pyrazole hybrids had an anticancer effect against four cancer cell lines. The findings showed that p‐methoxy hybrids displayed better cytotoxic effects than other thymol pyrazole hybrids due to the ether group on the thymol moiety. Thus, 5a and 5b hybrids were prominent as the most potent compounds against A‐549 and HT‐1080 cell lines (Laamari et al. 2024). Likewise, thymol hybrids 6a–m (1.8 μM) and 6b–f (1.4 μM) revealed greater antiproliferative properties than doxorubicin and 5‐FU (18.74 μM) against HCT‐116, MCF‐7, and HepG2 cell lines, indicating their better anticancer potential in comparison with drugs (Almalki et al. 2021). Thymol–ciprofloxacin hybrids were investigated against four cancer and one normal cell line. The compound 7a–b demonstrated a promising anticancer effect against the cancer cells with IC50 values (> 52 μM), compared to doxorubicin, and no substantial cytotoxic result was noticeable on the normal cell lines (Szostek et al. 2022).

Carvacrol hybrids were synthesized by Sisto et al. (2020) and evaluated against gastric adenocarcinoma cell lines. All the hybrids displayed poor activity compared to 5‐FU; however, the introduction of the benzyl moiety, specifically with 3‐CH3, 4‐SO2CH3, and 4‐SOCH3 on the meta‐ and para‐positions, upgraded the pharmacological activity of hybrid compounds. In another investigation, compound 8 of carvacrol hybrids was an effective anticancer compound with IC50 values of 0.47 and 0.75 μM against MCF‐12A and MCF‐7 lines, respectively. The better anticancer activity was credited to integrating artesunate by an ester link into the carvacrol moiety (Mbese et al. 2022). The derivatives/hybrids of thymol and carvacrol with anticancer potential are described in Table 4.

**TABLE 4:** Anticancer activity of thymol and carvacrol hybrids against different cancer cell lines.

Compound	Hybrids	Study type/cell lines	IC50/concentration	References
Thymol	3	PC‐3, MCF‐7, K562, and SH‐SY5Y	12.8 μM and 5.96 μM	Demirbolat et al. (2022)
	4	MCF‐7, A‐549, MDA‐MB‐231, and HT‐1080	7.10–19 μM	Laamari et al. (2023)
	5a and 5b	A‐549 and HT‐1080	22.17–62.72 μM	Laamari et al. (2024)
	6a–m, 6b–f	HCT‐116, MCF‐7, and HepG2	1.8 μM and 1.4 μM	Almalki et al. (2021)
	7a–b	SW480, HCT116, HepG2, HaCaT	> 52 μM	Szostek et al. (2022)
	11a	MCF‐7, PC‐3, and HT‐29	2.48 μM	Zengin Kurt et al. (2023)
	12a–c	PC3 and DLD‐1	7.67 μM and 12.39 μM	Sahin et al. (2023)
	14a–c	HepG2, A549, MCF‐7, and HeLa	6.24–11.96 μM	Yu et al. (2020)
Carvacrol	16, 21, 35, 38	Gastric adenocarcinoma	6.5 μM	Sisto et al. (2020)
	8	MCF‐7, MCF‐12A	0.47 and 0.75 μM	Mbese et al. (2022)
	9a–d	*Artemia salina* nauplii (Brine shrimp method)	50.39 μg/mL	Valverde Sancho et al. (2023)
	10a–c	SH‐SY5Y and HEK‐293	9.79–64.72 μM	Vasconcelos et al. (2024)
	11b	PC3, HT‐29, and MCF‐7	9.10, 9.40, and 12.01 μM	Zengin Kurt et al. (2023)
	15a–c	MCF‐7, MD‐MBA‐231, and HeLa	51.05–64.75 μg/mL	Khwaza et al. (2023)

## 10 Clinical Trials and Strategies to Increase the Therapeutic Efficacy

Clinical trials are research studies that evaluate new treatments, drugs, or medical interventions in humans to assess safety, efficacy, and side effects before regulatory approval for widespread medical use. Despite thymol and carvacrol's prime importance in cancer therapy, the clinical trials are lacking in anticancer perspectives, thus hindering their medicinal and clinical significance. However, some trials on other diseases are available, illuminating their therapeutic role in disease management. In a trial with 30 participants fed on thymol (25 mg/kg/day) orally for 30 days and gel application along with low‐level laser therapy, reduced cytokines (IL‐1β, TNF‐α), MDA, H_2_O_2_, triglycerides, and total cholesterol in patients with type 2 diabetes mellitus and dermatitis (Martirosyan et al. 2022). In a randomized, cross‐over study, 12 individuals were provided with two different dental varnishes, including 10% fluoride (F‐) varnish and 1% chlorhexidine (CHX) and 1% thymol varnish. The outcomes showed that both varnishes exhibited a better remineralization effect than control (Bizhang et al. 2015). The safety and tolerability of carvacrol were evaluated in healthy participants receiving 1 and 2 mg/kg/day for 1 month. The ESR, calcium, MCV, Hb, and HCT levels were decreased, while CPK, LDH, MCH, MCHC, and triglycerides increased in the 1 mg/kg/day group. The 2 mg/kg/day group depicted significant reductions in HDL, total bilirubin, RBC, and HCT. The outcomes of this phase I study concerning carvacrol's impact on healthy subjects displayed clinical safety and tolerability (Ghorani, Alavinezhad, Rajabi, and Boskabady 2021). A randomized, placebo‐double‐blind clinical trial involving 33 participants was divided into two groups: placebo and carvacrol group (1.2 mg/kg/day, *n* = 17). The carvacrol group was the asthmatic patients' group, which received prepared capsules for 2 months and 3 times/day with routine medications. The results showed that respiratory symptoms and OS markers substantially declined after a 2‐month treatment with carvacrol. The anti‐inflammatory and antioxidant effects of carvacrol make it a promising therapeutic agent for asthma (Ghorani, Alavinezhad, Rajabi, Mohammadpour, and Boskabady 2021). The sta bility and bioavailability of compounds are critical to achieving their maximum functional efficiency in different fields. They rapidly lose stability in unfavorable conditions and are mostly sensitive to light, temperature, oxygen, and pH. Likewise, thymol and carvacrol are volatile, and their stability is influenced by these factors (temperature, light, oxygen, and pH). Both compounds are stable under moderate circumstances, but prolonged exposure to heat, UV light, or air can lead to degradation (Ates and Yildiz 2025). The stability and bioavailability of compounds can be increased via nanoencapsulation/nanocarriers (Niaz et al. 2021).

Novel techniques, like liposomes or polymer‐based carrier encapsulation, are often applied to improve their stability in formulations. Moreover, maintaining neutral to slightly acidic pH levels can preserve their activity, making them effective in food preservation and pharmaceutical applications (Gong et al. 2021). Regarding this, Ates and Yildiz (2025) determined the stability of carvacrol in β‐cyclodext rin metal–organic frameworks (β‐CD‐MOFs). They concluded that the thermal stability of the carvacrol‐β‐CD‐MOFs complex improved by alterations in temperatures. Furthermore, the antioxidant capacity and solubility of the complex also improved, as demonstrated by the controlled release behavior of carvacrol. Additionally, nanoencapsulation of carvacrol with Eudragit and chia mucilage was evaluated, and it was found that carvacrol and Eudragit NPs preserved their nanometric dimensions for 180 days, while carvacrol and chia mucilage complex sustained this property for only 30 days (Tópor Nunes et al. 2024).

Protein‐based nanocarriers are an effective way to enhance thymol's stability and application performance. Rao et al. (2024) studied the synergistic role of succinylation amendment and ε‐polylysine in thymol‐loaded succinylated ovalbumin/ε‐polylysine nanogel. They reported that succinylation elevated the negative charges of OVA, and ε‐PL complexation enhanced electrostatic interactions and hydrogen bonding of the nanogel. The study's findings indicated that the nanogel showed exceptional thermal and pH stability. Another study reported that thymol‐lauric acid‐loaded casein NPs (ThyLA‐NPs) revealed better thermal and storage stability than simple thymol‐casein NPs. The better performance of ThyLA‐NPs is due to the high hydrophobic nature of the eutectic solvents (Ge et al. 2023).

Ultrasonication is an effective technique to enhance the stability of thymol and other bioactive compounds. The ultrasonication relies on the efficacy of the cavitation mechanism, which includes sinusoidal pressure fluctuations within the fluid, thus causing cavitation bubbles. Moreover, due to their hydrophilic nature, polysaccharide nano‐emulsions can improve stability and solubility (Mehta et al. 2022). Recently, Phyo et al. (2024) used ultrasonication to enhance the stability and physicochemical properties of thymol and cinnamaldehyde‐loaded chitosan NPs. The study findings showed that 400 watts of power elevated the performance of NC‐CH formulations, and NC‐CH‐400 displayed increased solubility. Moreover, the NC‐CH‐400 formulation exhibited maximum thermal stability, verified by the melting points, indicating superior storage durability. The nano‐capsules also displayed a smooth microstructure without surface cracking.

## 11 Limitations

Thymol and carvacrol face significant limitations in clinical application due to poor stability and low bioavailability. Their volatility and susceptibility to degradation reduce efficacy during storage and processing. In vivo, both compounds are rapidly metabolized and eliminated, limiting their systemic availability. Moreover, their hydrophobic nature hinders effective absorption. Clinical trials evaluating thymol and carvacrol remain limited, with most evidence stemming from in vitro or animal studies. This lack of robust human trials regarding cancer hampers conclusive therapeutic validation. Overcoming these limitations requires the development of improved delivery systems and more comprehensive clinical research to establish the efficacy and safety profiles.

## 12 Conclusion and Future Perspectives

Thymol and carvacrol exhibit substantial anticancer potential through multiple molecular mechanisms, including apoptosis induction, oxidative stress modulation, and anti‐inflammatory effects. Their ability to enhance chemotherapy and radiotherapy efficacy while reducing toxicity makes them promising adjuncts in cancer treatment. Additionally, their synergy with other bioactive compounds further amplifies their therapeutic benefits. As natural, biocompatible agents, thymol and carvacrol offer a novel approach to cancer management, potentially improving treatment outcomes. Moreover, drug‐resistant cancer cells limit treatment efficacy, but thymol and carvacrol offer solutions by modulating resistance mechanisms, enhancing drug sensitivity, and improving therapeutic outcomes. Future research should optimize their delivery, bioavailability, and clinical applications to harness their full potential in integrative oncology. Advanced drug delivery systems, such as nano‐formulations and encapsulation techniques, can improve solubility, controlled release, and targeted action. Further clinical trials are required regarding cancer to validate their efficacy, safety, and synergistic potential with existing therapies. Understanding their pharmacokinetics and mechanisms of action will facilitate their transition into clinical applications. These efforts could establish thymol and carvacrol as effective anticancer agents, paving the way for novel, natural‐based therapeutics.

## Author Contributions

**Ahmad Mujtaba Noman:** conceptualization (equal), writing – original draft (equal). **Muhammad Tauseef Sultan:** conceptualization (equal), writing – original draft (equal). **Shehnshah Zafar:** resources (equal), writing – original draft (equal). **Muhammad Maaz:** data curation (equal), writing – review and editing (equal). **Aimen Mazhar:** writing – original draft (equal). **Muzzamal Hussain:** investigation (equal), writing – review and editing (equal). **Muhammad Imran:** data curation (equal), resources (equal). **Ahmed Mujtaba:** validation (equal), visualization (equal). **Muhammad Tajammal Hussain:** data curation (equal), investigation (equal), writing – review and editing (equal). **Suliman A. Alsagaby:** visualization (equal), writing – review and editing (equal). **Waleed Al Abdulmonem:** data curation (equal), writing – review and editing (equal). **Muhammad Asif Khan:** data curation (equal), investigation (equal), methodology (equal). **Entessar Al Jbawi:** data curation (equal), investigation (equal), supervision (equal).

## Ethics Statement

The authors confirm that this manuscript adheres to all ethical standards.

## Conflicts of Interest

The authors declare no conflicts of interest.

## Data Availability

The data that support the findings of this study are available on request from the corresponding author.
